# Digital health literacy in Somali higher education: a public health agenda for navigating health misinformation and generative AI

**DOI:** 10.3389/fpubh.2026.1962463

**Published:** 2026-09-18

**Authors:** Abdukadir Mohamed Hassan

**Affiliations:** 1 Department of Critical Care Medicine, Dr. Sumait Hospital, SIMAD University, Mogadishu, Somalia; 2 Faculty of Graduate Studies and Research, Somali National University, Mogadishu, Somalia

**Keywords:** digital health equity, digital health literacy, generative artificial intelligence, health misinformation, higher education, public health, Somalia, university students

## Abstract

Digital health literacy is becoming a core public-health capability as health information is increasingly encountered through search engines, social media, digital services, and generative artificial intelligence (AI). University students are frequent users of these environments, yet digital access does not necessarily confer the ability to evaluate source credibility, recognize misinformation, judge the limits of AI-generated advice, protect personal data, or know when professional guidance is required. This Perspective argues that digital health literacy in Somali higher education should be treated as both an individual competency and an institutional public-health responsibility. Somalia is strengthening digital health and health-information systems, while direct evidence on digital health literacy among university students remains sparse. Rather than proposing another generic AI-literacy model, we outline an implementation agenda that connects critical health-information appraisal, misinformation resilience, responsible generative-AI use, privacy awareness, and safe escalation with cross-cutting conditions of equity, affordability, language accessibility, trusted institutional information, and faculty capacity. Universities can operationalize this agenda through curriculum integration, clear guidance for health-related AI use, strengthened library and public-health information pathways, and locally relevant evaluation. Research should move beyond self-report-only surveys toward contextually adapted measures, performance-based tasks, multi-institutional studies, and intervention evaluation. Building these capabilities alongside digital infrastructure can help make Somalia’s digital-health transition safer, more equitable, and more accountable.

## Introduction

Digital transformation is changing not only how health services are organized but also how people encounter, interpret, and act on health information. Search engines, social media, mobile platforms, telehealth services, and generative artificial intelligence (AI) increasingly sit between individuals and formal health systems. The World Health Organization (WHO) therefore frames digital transformation as a health-system and public-health issue rather than a purely technological one (1). In this environment, the ability to access a device or connect to the internet is only a starting condition. Safe engagement also requires the capacity to locate relevant information, assess its credibility, understand uncertainty, apply information appropriately, protect personal data, and recognize when digital information cannot substitute for professional care.

The concept has evolved from early eHealth literacy, commonly defined as the ability to seek, find, understand, appraise, and apply health information from electronic sources (2), toward broader digital health literacy that includes operational skills, navigation, evaluation of reliability, content creation, and privacy-related competencies (3). University students are an important population because higher education is simultaneously a learning environment, a setting for health promotion, and a pathway into professional and civic roles. Yet recent systematic reviews show that the university-student evidence base remains heavily dependent on cross-sectional, self-reported measures and contains a persistent gap between literacy assessment and tested interventions (4, 5).

This Perspective argues that digital health literacy in Somali higher education should be understood as a public-health capacity and equity requirement, not simply as an individual student’s technical skill. The central proposition is that universities should move beyond assuming that digital access implies digital readiness. They should deliberately cultivate critical health-information appraisal, misinformation resilience, responsible use of generative AI, privacy and data awareness, and safe escalation to authoritative or professional sources. The contribution is therefore not another generic AI-health-literacy framework. It is a Somalia-focused implementation agenda that links student competencies to institutional responsibilities and to the broader conditions needed for an equitable digital-health transition. The argument is informed by recent systematic reviews, WHO global and regional guidance, Somalia-specific health-information-system documentation, and selected empirical studies; it is not intended as a systematic evidence synthesis. This Perspective contributes a context-sensitive implementation agenda that translates established digital health literacy principles into actionable priorities for a fragile and resource-constrained setting, explicitly linking student competencies to institutional responsibilities and equitable digital-health outcomes.

## Digital access is not digital health literacy

The distinction between access and literacy is consequential. A student may use social media daily, search for health information frequently, or be comfortable with digital interfaces while still having difficulty determining whether a source is authoritative, whether a claim is supported by evidence, or whether online advice is appropriate for a specific health decision. Studies among university students in Germany and across East and South-East Asia have demonstrated that digital health-information seeking is common while critical evaluation of information quality can remain challenging (6, 7). A recent study in a resource-limited university setting in Ethiopia similarly illustrates that eHealth literacy is associated with patterns of online information use and contextual factors, reinforcing the importance of studying competencies within their local digital environment rather than treating internet use as a proxy for literacy (8).

The measurement problem is equally important. The eHEALS scale was an influential early instrument (2), and later tools such as the Digital Health Literacy Instrument expanded assessment to a wider range of internet-based tasks (3). However, the 2026 systematic review of university-student digital health literacy found substantial measurement heterogeneity and concluded that the field remains dominated by descriptive self-report and cross-sectional designs (5). Self-perceived confidence is useful but cannot establish whether a student can correctly verify a source, identify a misleading claim, compare conflicting information, or decide when an AI-generated answer requires clinical verification. For Somali higher education, measurement should therefore be treated as part of the implementation problem: what matters is not only whether students report confidence, but whether they can perform critical tasks safely in the information environments they actually use.

## Why Somali higher education is a public-health setting for digital health literacy

The policy context makes this agenda timely. WHO’s Digital health action plan for the Eastern Mediterranean Region, 2024–2028 identifies digital literacy, governance, secure infrastructure, and workforce capacity as regional priorities and assigns academic institutions explicit responsibilities to research digital health, train the next generation of digital-health professionals, and integrate digital health into curricula (9). In Somalia, WHO’s comprehensive assessment of the national health information system recommended development of an e-health strategy, data-security and privacy legislation, governance for digital-health standards and interoperability, digital-literacy training, and competency-based curricula to sustain digital platforms (10). These recommendations show that human capacity and institutional governance are inseparable from technological deployment.

Higher education can contribute to this capacity in two ways. First, health-professions programs prepare future clinicians, nurses, public-health practitioners, and other workers who will increasingly interact with digital systems and with patients who bring online information into care encounters. Second, universities educate a much wider population of digitally engaged adults whose health decisions, peer networks, and community roles extend beyond the campus. A university-wide public-health approach can therefore establish a common foundation in source evaluation, misinformation recognition, privacy, and responsible AI use, while health-professions programs add discipline-specific competencies related to clinical risk, patient communication, and professional accountability.

Somalia-specific evidence should nevertheless be interpreted cautiously. A 2025 study among 219 health-sciences students at one university in Mogadishu assessed general health literacy and reported problematic-borderline scores on the instrument used (11). This is relevant background, but general health literacy is not equivalent to digital health literacy and cannot be used to infer students’ competence in evaluating online or AI-generated information. Likewise, a 2025 community survey in Mogadishu identified public concerns related to telehealth reliability, quality, and data privacy (12). These findings indicate that questions of trust and digital-health readiness are locally relevant, but they do not establish the digital health literacy level of Somali university students. Published evidence directly characterizing that construct remains sparse, which is precisely why policy and educational action should be paired with careful local measurement rather than assumptions.

## Misinformation and generative AI change the literacy challenge

Health misinformation is not new, but digital platforms can accelerate its production, repetition, and reach. WHO describes an infodemic as an overabundance of information, including false or misleading information, that can create confusion, harmful behavior, and mistrust (13). For students, critical digital health literacy therefore requires more than locating information quickly. It requires examining who produced a claim, what evidence supports it, whether the content is current, how uncertainty is communicated, and whether commercial or algorithmic influences may shape visibility.

Generative AI adds a different problem: information can be conversational, personalized in tone, and linguistically fluent without being clinically reliable. Reviews of large language models used for plain-language medical information identify meaningful opportunities for accessibility and explanation, but also risks related to accuracy, relevance, bias, transparency, privacy, and inappropriate reliance (14). WHO’s ethics guidance for AI in health similarly emphasizes human autonomy, transparency, accountability, inclusiveness, and safety (15). These considerations are particularly important when students move from using AI to summarize or translate information toward using it to interpret symptoms, medications, treatment choices, or urgent decisions.

A recent Frontiers Perspective has already advanced AI Health Literacy through a reflective framework centered on task appropriateness, context of use, and critical verifiability (16). A Somalia-focused Perspective should build on rather than duplicate that conceptual work. The practical implication for universities is to teach students to ask whether an AI tool is appropriate for the task, whether the context increases vulnerability to error or over-trust, and whether the answer can be checked against traceable evidence or professional guidance. Responsible use also includes protecting personal and patient information, recognizing uncertainty, and understanding that fluent output is not proof of medical validity.

## Digital health literacy is also an equity and institutional responsibility

An exclusively individual-skill model risks blaming students for limitations created by the environment around them. Digital health equity scholarship has long emphasized that digital innovation can reproduce existing social gradients when access, usability, trust, and skills are unevenly distributed (17). More recent work on the digital determinants of health broadens this argument by treating digital systems, data practices, connectivity, commercial platforms, and governance as conditions that can shape health opportunities and disparities (18). In higher education, digital health literacy should therefore be understood as the interaction between what a student can do and what institutions make possible.

For Somali universities, relevant enabling conditions include affordable and reliable connectivity, accessible devices and learning spaces, health information in language and formats that students can use, clear privacy protections, trustworthy institutional sources, and faculty who are prepared to teach critical appraisal in contemporary digital environments. The agenda should also accommodate students with differing levels of prior digital exposure rather than assuming a uniform baseline. This matters because an educational intervention that teaches source appraisal but ignores connectivity costs, inaccessible resources, or lack of trusted local information can widen rather than narrow disparities. Because trust and authority are culturally situated, local implementation should also examine how students weigh institutional guidance alongside family, religious, traditional, and community sources before defining “trusted” information pathways; locally credible actors may be engaged where appropriate while preserving evidence-based standards and avoiding assumptions that any source is uniformly trusted.

Institutional responsibility is equally important for generative AI. Universities need clear guidance that distinguishes acceptable educational uses from health-related uses that carry greater risk. Policies should not be limited to academic integrity or plagiarism. They should also address privacy, entry of sensitive information into external systems, verification of health claims, citation of source material, and escalation when AI output could influence diagnosis, medication, treatment, or emergency decisions. In this sense, digital health literacy is not a one-time student workshop; it is an organizational capability involving curricula, libraries, information technology, student health services, faculty development, and public-health leadership.

## A public-health agenda for Somali higher education

A practical agenda should begin with curriculum integration rather than a stand-alone digital-literacy course. Foundational competencies can be embedded in first-year orientation, information-literacy teaching, public-health and research-methods courses, and health-professions curricula. Students should repeatedly practice distinguishing authoritative from non-authoritative health sources, triangulating claims, identifying uncertainty, checking publication dates and provenance, and recognizing when online information is insufficient for safe action. A recent scoping review of higher-education misinformation interventions identified media- and information-literacy approaches as a recurring strategy while also highlighting heterogeneity in intervention designs and outcomes (19). Repeated, contextually relevant practice is therefore preferable to assuming that a single lecture produces durable competence.

Second, universities should create trusted information and verification pathways. Libraries, faculties, and student health or public-health units can curate links to authoritative national, regional, and international health sources and teach students how to move from a social-media post or AI response to the underlying evidence. The aim is not to prohibit digital platforms but to make verification easier and routine. Where reliable local content is limited, institutions can support the development or adaptation of accessible health information while clearly distinguishing verified guidance from educational material or general-purpose AI output.

Third, responsible generative-AI use should be taught as a safety practice. Students should understand that appropriate use depends on the task, the stakes of the decision, the availability of verification, and the sensitivity of information entered. Low-risk educational uses such as generating study questions or simplifying non-sensitive text are different from asking an AI system to diagnose symptoms or recommend treatment. Health-professions programs should go further by linking AI literacy to professional duties, evidence-based practice, informed communication, and protection of patient information.

Fourth, equity and privacy should be designed into implementation. Universities should monitor whether digital-health learning activities unintentionally disadvantage students because of device ownership, connectivity cost, disability, language, or prior digital experience. Alternatives should be available where required, and institutional procurement or platform adoption should consider accessibility and data governance. Privacy education should include practical attention to what information should not be entered into public AI tools, how digital traces are created, and why convenience does not remove obligations to protect sensitive information.

Finally, implementation should be evaluated. Figure 1 presents a staged agenda that moves from access through navigation, appraisal, misinformation resilience, responsible AI use, and safe health action, while treating equity, language accessibility, privacy, trusted sources, faculty capacity, and evaluation as cross-cutting conditions. It is deliberately an implementation agenda rather than a new validated literacy model. Table 1 translates the argument into institutional actions and measurable evidence needs. Universities can start with feasible pilots, but expansion should depend on what students can actually demonstrate and on whether interventions reduce rather than reproduce disparities.

**Figure 1:**
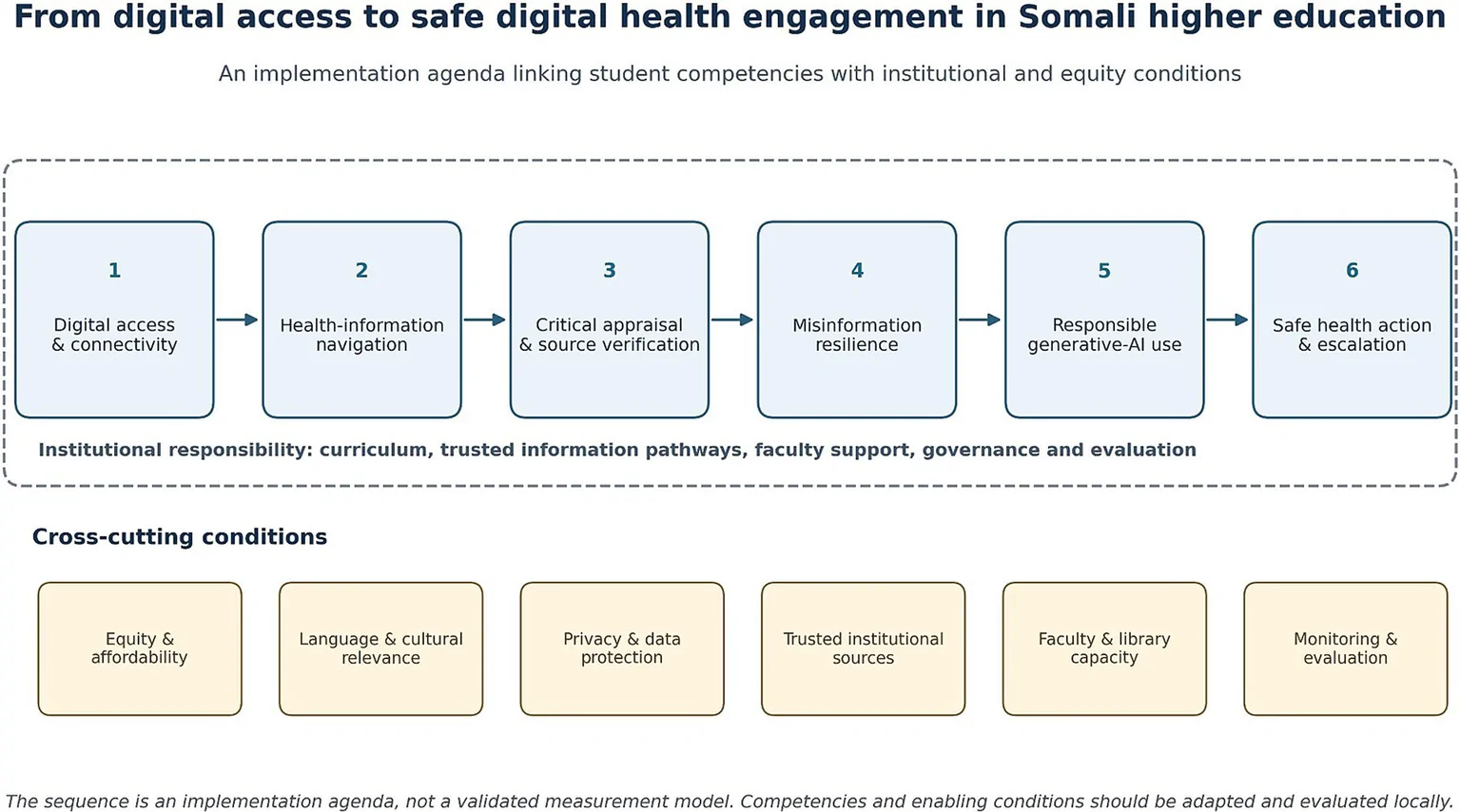
From digital access to safe digital health engagement in Somali higher education. The figure presents a staged implementation agenda linking digital access, health-information navigation, critical appraisal, misinformation resilience, responsible generative-AI use, and safe health action with cross-cutting conditions of equity and affordability, language and cultural relevance, privacy and data protection, trusted institutional sources, faculty and library capacity, and monitoring and evaluation. Arrows indicate a practical learning and implementation sequence, not causal relationships or a validated developmental or measurement model.

**Table 1:** Priority actions for strengthening digital health literacy in Somali higher education.

Priority domain	Proposed institutional action	Public-health rationale	Suggested indicators/evidence needs
Curriculum and competencies	Embed source appraisal, misinformation recognition, privacy, and responsible AI use in orientation, information-literacy, public-health, research-methods, and health-professions teaching.	Moves beyond digital access toward repeated practice in health-information judgment.	Verification-task performance; curriculum coverage; competency change.
Trusted information pathways	Create library- and faculty-supported routes from social-media or AI claims to authoritative national, regional, and international sources.	Makes verification easier and reduces reliance on source popularity or fluency.	Use of curated resources; source-selection accuracy; trust and usability.
Generative-AI safety	Differentiate low-risk learning uses from higher-risk health decisions; teach verification, uncertainty, escalation, and restrictions on sensitive data entry.	Addresses the gap between fluent output and medically reliable information.	Appropriate-use decisions; verification; privacy-safe prompting; escalation.
Equity and accessibility	Design activities around connectivity cost, device access, disability, language accessibility, and prior digital experience; provide alternatives where needed.	Prevents literacy initiatives from reproducing digital disparities.	Participation and outcomes by access characteristics; accessibility audits; unmet needs.
Privacy and governance	Align student guidance with institutional data protection, platform governance, academic integrity, and health-safety policies.	Treats digital health literacy as an organizational rather than solely individual responsibility.	Policy availability and awareness; privacy practices; governance review.
Faculty, library, and public-health capacity	Prepare educators and librarians to teach contemporary digital appraisal and coordinate with student health/public-health services.	Builds sustainable institutional capability and trusted support.	Faculty-development uptake; cross-unit activity; use of support services.
Evaluation and research	Use contextually adapted validated measures with task-based assessments; test interventions and monitor equity of benefit.	Addresses self-report and literacy-to-intervention gaps in the current evidence base.	Reliability/validity evidence; task performance; intervention effect; subgroup equity.

## From agenda to evidence: research priorities

The immediate research priority is to characterize digital health literacy in Somali higher education without reducing it to a single self-reported score. Multi-institutional studies should combine validated or carefully adapted instruments with task-based assessment of source verification, misinformation appraisal, privacy decisions, and appropriate use of AI-generated health information. Measures should undergo linguistic and contextual adaptation where necessary, and studies should report reliability and validity evidence rather than assuming that an instrument validated elsewhere performs identically in Somalia.

Research should also examine the distribution of competencies. Potentially relevant factors include field and year of study, prior training, internet access and affordability, preferred information sources, use of social media for health information, use of health applications, and experience with generative AI. Regional evidence, including a 2024 Ethiopian university study, illustrates why digital environment and information-seeking behaviors deserve attention in resource-limited settings (8). However, predictors identified elsewhere should be treated as hypotheses for Somalia rather than presumed determinants.

Most importantly, the research program should progress from description to intervention. The 2026 systematic review identified a clear literacy-to-intervention gap and called for objective competence assessment and theory-informed trials embedded in university systems (5). Somali studies could test brief verification modules, library-led information-literacy sessions, curriculum-integrated AI safety activities, or structured exercises comparing social-media, search-engine, and generative-AI health claims. Outcomes should include demonstrated appraisal skills, appropriate escalation decisions, and equity of benefit across student groups, not only satisfaction or self-confidence.

## Discussion

Digital health literacy has become more consequential as health information moves across social media, digital services, and generative AI, but the central challenge is not technological novelty alone. It is whether institutions develop the human capabilities and safeguards required for people to use these environments safely. For Somalia, this question intersects with an ongoing digital-health and health-information-system transition in which governance, privacy, interoperability, and workforce capacity are already recognized priorities (9, 10). Higher education is therefore a plausible implementation setting for connecting individual competencies with institutional public-health responsibility.

This Perspective has limitations. Somalia-specific empirical evidence directly measuring digital health literacy among university students is sparse, and the proposed agenda necessarily draws on a combination of national and regional policy documents, limited adjacent Somali evidence, and international research. The agenda should consequently be treated as a set of evidence-informed priorities to be adapted and tested, not as proof of existing student deficits or as a validated Somali competency framework. It also does not assume that generative AI is uniformly beneficial or harmful; the appropriate response depends on task, context, verifiability, governance, and access. The proposed agenda is a theoretical and practical synthesis: its constituent components are evidence-informed, but their comprehensive implementation in Somali higher education remains untested. It should therefore be treated as a testable hypothesis for institutional change rather than as an established best-practice guide.

Somali universities have an opportunity to build digital health literacy before digital adoption outpaces the capacity to evaluate and use health information safely. The priority is not merely to increase access, but to create learning environments in which students can verify claims, resist misinformation, use generative AI with appropriate caution, protect sensitive information, and recognize when authoritative or professional guidance is required. Embedding these capabilities in curricula, institutional information pathways, faculty development, and evaluation can make digital transformation more equitable and accountable while generating the local evidence needed for future policy and practice. The implementation logic may also be relevant to other fragile or resource-constrained higher-education settings in which digital adoption is advancing faster than local evidence, institutional guidance, and safeguards, although transfer requires contextual adaptation.

## Data Availability

No new datasets were generated or analyzed for this Perspective. All evidence discussed is drawn from the published literature and publicly available policy or guidance documents cited in the article.
